# Passive Infrared (PIR)-Based Indoor Position Tracking for Smart Homes Using Accessibility Maps and A-Star Algorithm

**DOI:** 10.3390/s18020332

**Published:** 2018-01-24

**Authors:** Dan Yang, Bin Xu, Kaiyou Rao, Weihua Sheng

**Affiliations:** 1 School of Information Science and Engineering, Northeastern University, Shenyang 110819, China; yangdan@mail.neu.edu.cn; 2 School of Physics, Northeast Normal University, Changchun 130000, China; raoky290@nenu.edu.cn; 3 School of Electrical and Computer Engineering, Oklahoma State University, Stillwater, OK 74074, USA; weihua.sheng@okstate.edu

**Keywords:** smart home, indoor position tracking, PIR sensors, accessibility map, A-start algorithm

## Abstract

Indoor occupants’ positions are significant for smart home service systems, which usually consist of robot service(s), appliance control and other intelligent applications. In this paper, an innovative localization method is proposed for tracking humans’ position in indoor environments based on passive infrared (PIR) sensors using an accessibility map and an A-star algorithm, aiming at providing intelligent services. First the accessibility map reflecting the visiting habits of the occupants is established through the integral training with indoor environments and other prior knowledge. Then the PIR sensors, which placement depends on the training results in the accessibility map, get the rough location information. For more precise positioning, the A-start algorithm is used to refine the localization, fused with the accessibility map and the PIR sensor data. Experiments were conducted in a mock apartment testbed. The ground truth data was obtained from an Opti-track system. The results demonstrate that the proposed method is able to track persons in a smart home environment and provide a solution for home robot localization.

## 1. Introduction

The elderly population is rapidly increasing worldwide. In 2050, the number of elderly people (60 and above) is forecast to reach a staggering 2 billion [1]. For personal comfort and due to limited medical resources, most of them live alone in their own houses instead of nursing homes. Elders usually prefer to stay in the comfort of their home where they feel more confident than moving to an expensive adult care or healthcare facility. Hence, how to help them to live conveniently and safely has become an important social issue [2]. Real-time indoor human monitoring plays an important role in assisted living, emergency detection, among many other aspects. A fundamental problem in human monitoring is how to localize humans in indoor environments.

Researchers have developed various techniques to localize residents in indoor environments [3]. Active Badge [4] was developed at the Olivetti Research Laboratory using diffuse infrared technology. It employs a network of sensors placed around the building with each room having at least one sensor. The subjects must carry a tag that can emit a unique code for approximately a tenth of a second every 15 s (a beacon) in order for the sensors to detect it. A master station, also connected to the network, polls the sensors for badge detections, processes the data, and then makes it available to clients that can display it in a useful visual form. When a sensor receives signals from the tag, the human was known to be in that room. Cricket [5] is an example that uses ultrasound technology to localize people. The Cricket indoor location system consists of location beacons and listeners. Beacons are transmitters attached to the ceiling of buildings and receivers called listeners are attached to the devices (or people) requiring location information. Active beacons transmit location information and an ultrasonic pulse. The passive listeners capture this information to calculate its distance from the beacon. This method would work for a large office environment. In [6], WiFi Received Signal Strength (RSS) fingerprints were used to detect persons’ location and estimate their velocity. Signal strength attenuation will cause lower localization accuracy. In [7], the authors’ solution automatically identifies the rooms where the smart objects are placed, by using the information contained within the RSS, the knowledge of the map of the house and the use of a fuzzy inference system without any a priori knowledge of the positions of the anchor nodes (ANs). In [8] multiple cameras and floor sensors were used to localize a human in a smart home environment. The system consisted of three components: camera localization, sensory floor localization and condensation tracker. The presence of a person was detected by the sensory floor by measuring the pressure variation. The cameras detected the person by using background subtraction and human template matching. Obviously, a large number of sensors will increase the cost of installation and there are always privacy concerns when cameras are used. In [9], they used ultrawideband (UWB) technology for indoor positioning. They presented an IPS based on UWB, which consists of four fixed transmitters and some mobile users. The transmitters, which are synchronized using a time division scheme, send a UWB signal to the mobile users. The estimation of location was calculated using a triangulation method based on the measure the Time Difference of Arrival (TDoA) between transmitters and receivers. In [10], they make used of Earth’ natural magnetic field orientation to perform the localization process. They measures location using disturbances of Earth’s magnetic field caused by structural steel elements in a building. Magnetic maps are developed taking measurements along corridors and landmarks and then places identified based on the magnetic signature. Ojeda et al. [11] developed a Zero Velocity Update (ZUPT)-based method that compensates the accelerometer bias and achieves an error of about 2% for short walks. These aforementioned methods all have their own limitations when providing a practical personal-tracking solution. 

Recently, passive infrared (PIR) sensors have been used to detect and localize humans because of their simplicity and less privacy concerns. Luo and Chen [12] proposed a technique to track the path of a human using PIR sensors. An array of PIR sensors was placed in a room so that the sensing areas of two or more sensors overlap. The experiment was conducted on a test bed in which a total of 12 sensors are placed to ensure the maximum overlap and the full coverage of the test area. A location accuracy of 0.5 m was achieved by the system. The accuracy of these PIR-based location systems greatly depends on the distribution density of sensors and the intersection of the field of views (FOVs) of the sensors. To reduce sensory uncertainty, multi-sensor techniques and data fusion algorithms are usually adopted. In [13], Yang et al. developed a low-cost and small-size human tracking system based on PIRs and wireless sensor network. A localization method based on detecting angle bisectors of PIR sensors and data fusion was presented. Then, Kalman filtering and particle filtering were employed for target tracking. In [14], Al-Naimi presented an automatic identification and tracking method by combining data from PIR sensors and floor pressure sensors. In [15], a method using PIR and RF for localization was proposed to deal with the RSS variation issue that caused a significant localization error. PIRs were used to identify the rough area, then the position of the person was estimated by applying the K Nearest Neighbor (K-NN) algorithm to the fingerprints inside that area. That method can reduce the localization error. In [16], they proposed a region-based human tracking algorithm based on the output signals of several PIR sensors. A mathematical abstraction of a PIR sensor as a building block for the algorithm is provided. In [17], a human indoor localization system based on ceiling mounted PIR sensor nodes was proposed. In the system, five sensor nodes are utilized to form a wireless sensor network (WSN). The spatial information is embedded in the PIR signal triggered by the human movement and is decoded based on the coding scheme. The Kalman Filter and the Kalman Smoother are used to refine the location estimation of the human target. 

In this paper, we propose a new solution to PIR-based indoor human localization by using an A-algorithm and a grid-based accessibility map. In our solution, the grid-based accessibility map is built which reflects human evaluation of the degree of accessibility in a smart home. We distinguish these areas based on obstacles (walls), furniture, unoccupied free space, accessibility-related daily habits, and so forth. PIR sensors data give a rough estimate of the human location without capturing any user images. They are deployed according to the area accessibility as the standard. To further improve the accuracy, we also exploit the A-star algorithm for indoor human tracking. First, the continuous triggering of two PIR sensor areas are defined as the given initial node and goal node of A-star algorithm. The weight function will be as the path-cost evaluation function. Eight candidate state nodes are selected around the current initial node. Their weight coefficients are calculated according to their direction and distance related the current initial node, and corresponding area accessibility value. The target node is the maximum of the weight function. The A-star algorithm fuse the PIR sensor data and grid-based accessibility map information to obtain more accurate position estimate. The rest of the paper is organized as follows: Section 2 details our tracking methodology, including how to build an accessibility map for a specific home, the details of the A-star algorithm for tracking and the PIR sensor model. Section 3 will present the experiments results. Section 4 concludes this paper and suggests future work. 

## 2. Methodology

### 2.1. Grid-Based Accessibility Map Building

**Figure 1:**
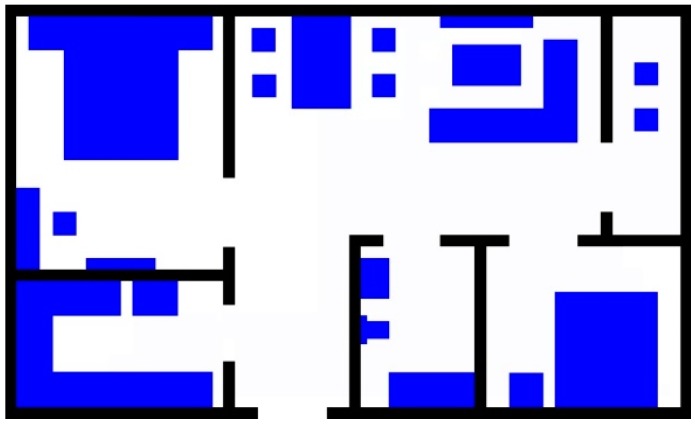
The floor plan of the mock apartment.

In this paper, we use a grid-based accessibility map to model the tracking environment. The grid-based accessibility map is subdivide into a finite position grid for the given environment. Each grid provides two pieces of information. The first one is the location of the grid in the environment and the second one is its probability of a person being present at that location. Detailed explanations are presented in the following paragraphs. A mock apartment (12 × 7.2 m^2^) used to test our ideas, as shown as Figure 1. The function A_map(x,y) is defined for describing the accessibility value at the location (*x*, *y*) in 2D *x*-*y* plane of the given environment.

**Figure 2:**
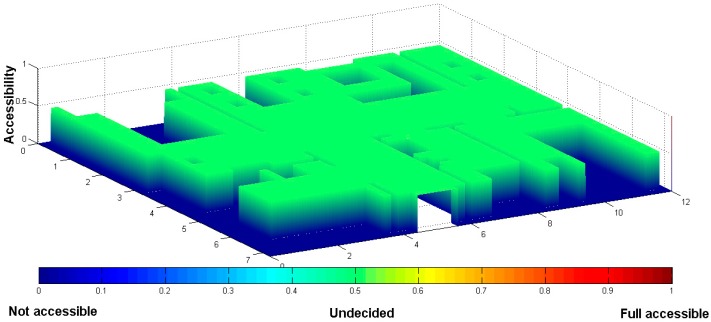
The initial value of the $A\_map(x_{t},y_{t})$.

The different colors are used to express different regions. The blue areas and the black areas denote the furniture and the walls, respectively. These areas cannot be walked in, and $A\_map(x_{t},y_{t})$ is set as “0”. Free space areas have no obstacle for access, so we store “0.5” as the original value. The original two-dimensional floorplan is converted into a 3D depth map according to the furniture layout, as shown as Figure 2. 

**Figure 3:**
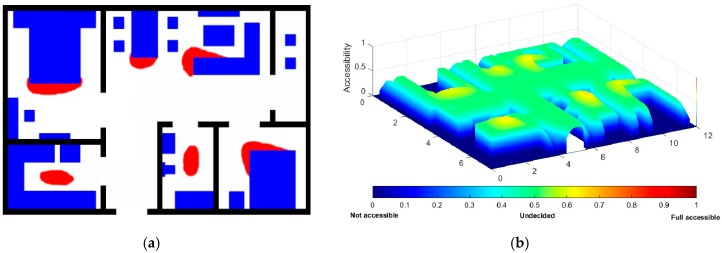
Hot areas related with the furniture layout: (**a**) hot areas related with furniture; (**b**) after processing with the Gaussian function.

To build the accessibility map, we use another source of information, i.e., the daily visiting habits of human subjects when they interacts with the environment. Generally, the human subject visits more frequently around certain furniture. The mock smart home we designed has some key furniture: bedroom bed, living room couch, dining room chair, living room table, kitchen basin and bathroom basin. The furniture locations in the indoor environment are known. The areas around the furniture has more visiting probability than other inner areas in the indoor environment, as shown in Figure 3a. Applying a Gaussian kernel also yields smooth transitions between the more visited areas and the surrounding areas, as described in Figure 3b.

**Figure 4:**
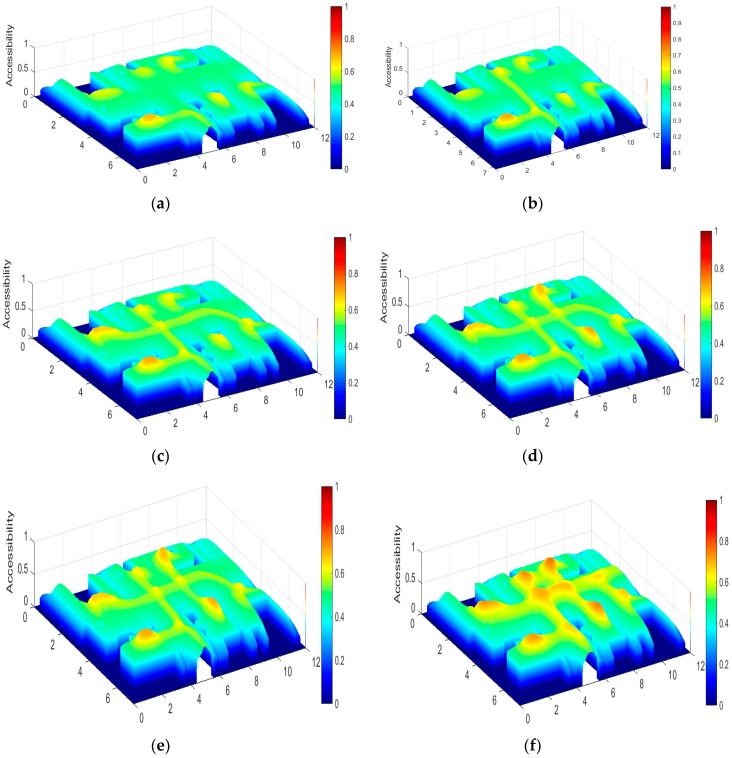
The accessible area heat map with several training. (**a**) result with p1 training; (**b**) result with p2 training; (**c**) result with p3 training; (**d**) result with p4 training; (**e**) result with p5 training; (**f**) added results with p1-p5 40 times training.

Besides these considerations, each two landmarks can generate an arbitrary route. The visiting probability of some routes is based on the observations about the specific person’s daily habits. The starting and ending landmarks can be (p1) entrance to kitchen, (p2) kitchen to living room table, (p3) major bedroom to guest room, (p4) living room sofa to bathroom, and (p5) guest room to balcony as shown in Figure 4. Tests with different trajectories were processed. The areas in these trajectories have more accessible frequencies than other areas of the apartment. These values can be obtained by observation.

**Figure 5:**
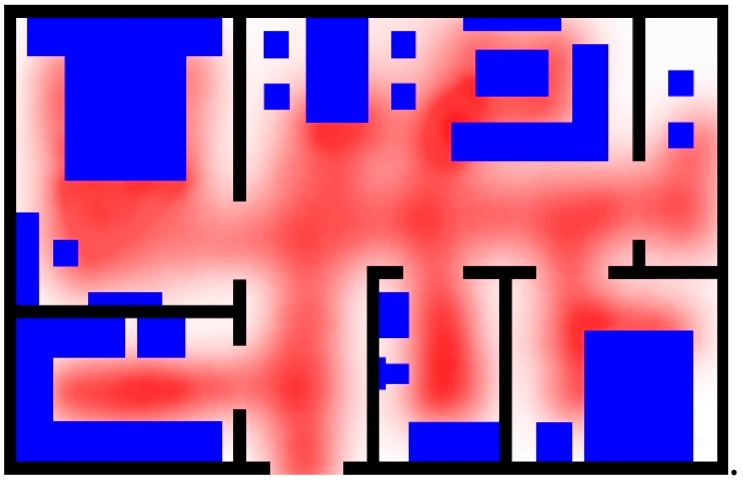
The accessible map considering the daily visiting route.

After all these processing steps, the accessiblity map in 2D view is shown in Figure 5.

**Figure 6:**
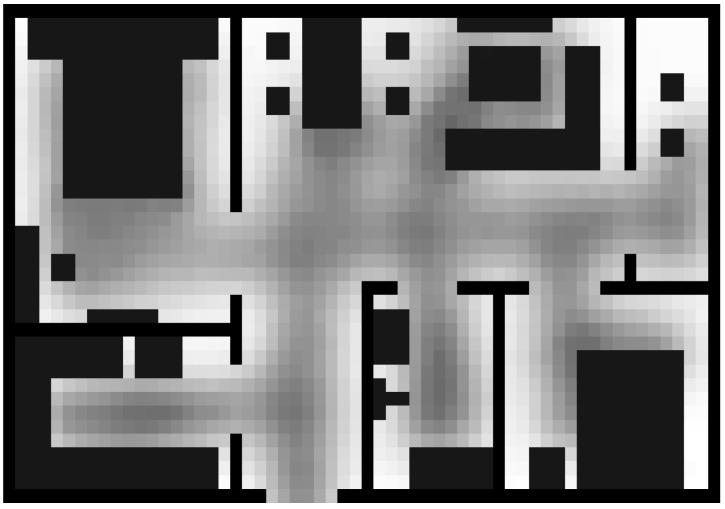
The grid-based grey image of accessible map.

Based on this prior knowledge, the accessibility map can be represented as a heatmap of the counts of the times that the assumed occupant visits respective grid nodes. Each grid size is Δx×Δy, and Δ*x* = Δ*y* = 0.1 m. The binary image of accessible map is shown as Figure 6. Let *N* denote the number of visitable grid nodes, and *h_i_* denotes the “heat” score of the *i-*th grid node. Therefore the accessibility map can be defined as a set of values $\{h_{1},\ldots ,h_{N}\},0<h_{i}<255$. 

### 2.2. Algorithm Design

The A-Star algorithm uses the method of best-first search and finds an optimal path from the initial node to the destination node [18]. It uses a distance and a cost heuristic function (usually denoted by f(n)) to determine the order in which the search visits nodes in the tree. The distance-plus-cost heuristic is a sum of two functions: (i) the path-cost function, which is the cost from the starting node to the current node (denoted by g(n)) and (ii) an admissible “heuristic estimate” of distance to the goal (denoted by h(n)):

(1) f(n)=g(n)+h(n)

The A-Star algorithm creates a tree of nodes and maintains two lists, an OPEN list and a CLOSED list. The OPEN list is a priority queue and keeps track of those nodes that need to be examined, while the CLOSED list keeps track of nodes that have already been examined. Each node *n* maintains f(n); intuitively, this is the estimate of the best solution that goes through *n*. If the initial node and node *n* are combined into one node, then the cost function is:

(2) f(n)≈h(n)

**Figure 7:**
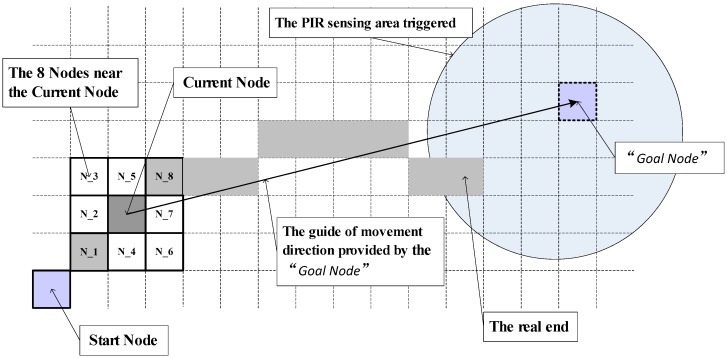
Diagram of target node estimation by the A-star algorithm.

Figure 7 shows the principle of next position estimation based on the A-star algorithm for tracking in the given smart home. The start node is denoted as the original position at the entrance of the room. At time *t*, the person is located at the current node. The blue circle area is the current trigger PIR sensing area, the “Goal Node” is the node with maximum heat score of the current trigger PIR sensing area. The eight candidate nodes N_1~N_8 for time t+1 are selected around the current node. The target node is determined by the following equation:

(3) $$h(x_{n},y_{n})=w_{dir}\cdot w_{h\_d}\cdot \frac{A\_map(x_{n},y_{n})}{\sqrt{{\left(x_{n}-x_{Goal}\right)}^{2}+{\left(y_{n}-y_{Goal}\right)}^{2}}},n=1\sim 8$$

where $A\_map(x_{n},y_{n})$ denotes the “heat” score for the candidate node *n* of position $\left(x_{n},y_{n}\right)$ obtained from the grid-based accessibility map. The goal node $\left(x_{Goal},y_{Goal}\right)$ is the node with the maximum heat score of the triggered PIR sensing area. The weight coefficient $w_{h\_d}$ represents the effect of heat score and distance between the target node and goal node. The direction weight $w_{dir}$ is determined by the relative position between the eight candidate nodes with the current estimated direction. For the time of no PIR sensor triggered, the estimated direction is from the current node to the point with maximum “heat” score in the next triggering PIR sensor area. For the time of one or two PIR sensors are triggered, the estimated direction is from the current node to the point with maximum “heat” score in the current triggering PIR sensor area during the first half part time of PIR sensor triggered. During the latter half part of the time the PIR sensor is triggered, the direction is from the current node to the point with maximum “heat” score in the next triggering PIR sensor area. Suppose that the subject who is tracked moves at a specific velocity, then the time of the tracked route can be denoted as a grid number in the grid-based accessibility map.

### 2.3. PIR Sensor Model

**Figure 8:**
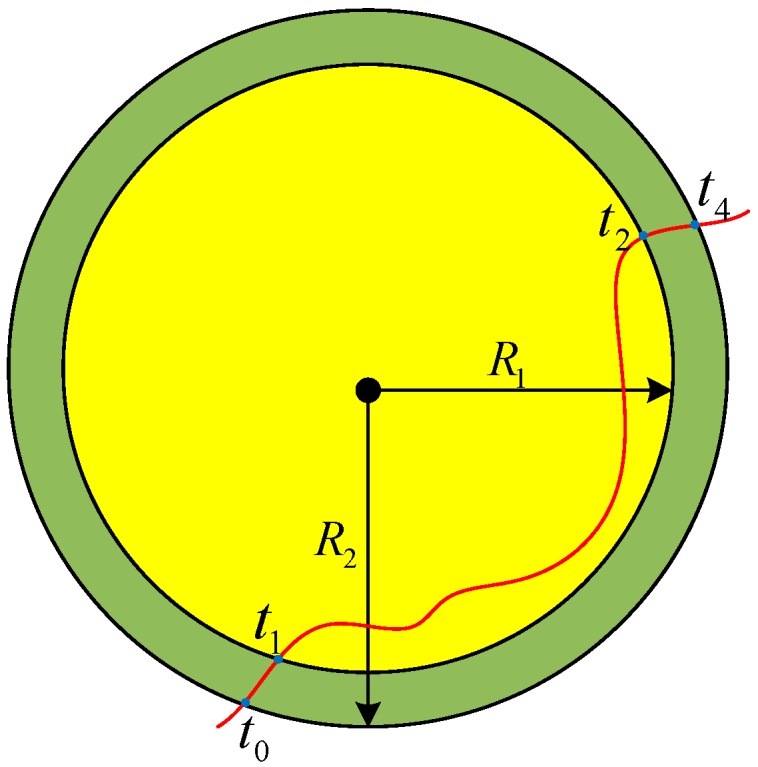
PIR Sensor Model.

In the ideal PIR sensing model, a PIR sensor reports “detected”, which means there is a person within the sensor detection region (the circle region with the radius *R* of the PIR sensor). For a practical PIR sensor, there is a time delay when a PIR sensor detects the target, so we define a more practical model of the PIR sensors as shown in Figure 8.

A person is always detected within an inner disk of radius *R*_1_ when moving in the sensor’s detection area, while the PIR sensor physical sensing area is the outer disk of radius *R*_2_. Time *t*_0_–*t*_4_ represents the time of human in moving. Time *t*_0_ to *t*_1_ and *t*_2_ to *t*_4_ the person has already been in the PIR sensor area, but the PIR sensor reports “no person”. Only from time *t*_1_ to *t*_2_, does the PIR sensor data reflect a “true “measurement.

## 3. Experiments and Results

### 3.1. PIR Sensor Parameters

**Figure 9:**
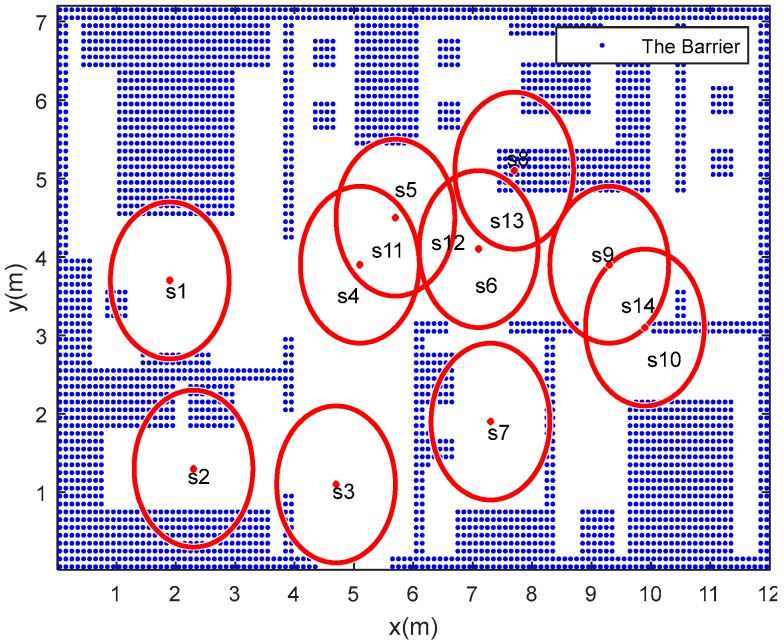
PIR deployment and sensing area.

The x-y plane area of the given smart home environment is 12 m × 7.2 m^2^. The thickness of wall is 0.2 m, the door of the kitchen and bathroom space is 0.2 × 1.0 m^2^, and other door spaces are 0.2 × 1.0 m^2^. The total possible coverage area for the PIR sensors is 75.12 m^2^. The number and deployment of PIR sensors are defined according to our previous work [19]. PIR sensor parameters are listed in Table 1. For the free space in the smart home environment, the degree of accessibility for a given area is obtained by training. The whole apartment floorplan is gridded by 8640 points. The distance between two points is 0.1 m. The blue areas represent the obstacles. The PIR sensor deployment, room layout in 2D view and PIR sensing areas are shown in Figure 9.

### 3.2. Hardware Platform

**Figure 10:**
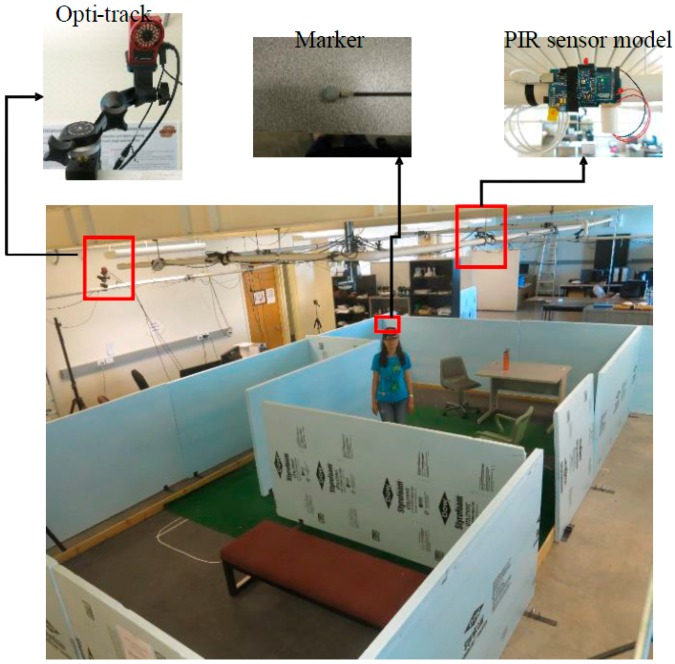
Testbed with Opti-track System and PIR sensors.

The testbed contains a motion capture system that provides the ground truth of human location, shown in Figure 10. A human subject wearing a cap with reflective markers on it was asked to move around the mock apartment, enabling the head location to be tracked by the OptiTrack system. This is the OptiTrack system manufactured by Natural Point Inc (Corvallis, OR, USA). [16] and it has 12 V100:R2 cameras. Special reflective markers are placed on the human body to track human subjects. We used EKMC1601111 units (Panasonic Electric Works, Tokyo, Japan) as the PIR sensor cells. A hand-made cylindrical lens hood was used to narrow the detection area of each sensor. The PIR data was read every 0.05 s from each sensor (sampling rate of 20 Hz). The radius of the narrowed PIR sensing area is less than the PIR sensing inner radius *R*_1_. This can offset the effect of PIR sensor response time. A ZigBee module is used for transmitting the PIR sensor data. Based on these data, the server PC estimates the human’s location in the apartment and derives the movement trajectory of the human.

### 3.3. Human Tracking

To evaluate our method, we pre-defined two trajectories. For route1, the human subject entered the apartment triggering the PIR at the front door and the system started performing localization and tracking. He is asked to have a rest at the couch, than go to the bedroom to change clothes, and finally he goes to the other bedroom to relax. For the route2, the person is coming home after shopping, and he first goes to the kitchen for preparing the meal, then has a dinner at the table at the living room, and at last goes to the bathroom and washes his hands. 

**Figure 11:**
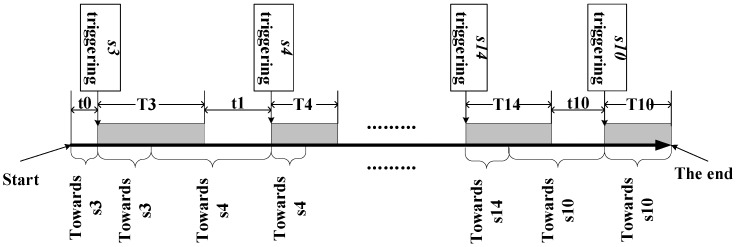
State transition for the route1.

For the route1, the beginning of the route is the “Start Node’, the Goal Node is the point with maximum heat score in the PIR sensor area (s3). According to Equation (3), the cost function *h*(*n*) value of the eight candidate estimated points are calculated. The next moment position is the point with the maximum *h*(*n*) value. T3 denotes the triggering time of the PIR sensor (s3). During the first half time of T3, “Start Node” is still the begining of the route, “Current Node” is the last estimated point of the route, and “Goal Node” is still the point with maximum heat score in the PIR sensor area (s3). For the latter half time of T3 and t1, “Start Node” changes to the point with the maximum heat score in the PIR sensor area (s3). “Goal Node” is still the point with the maximum heat score in the PIR sensor area (s4). In our lab, suppose that the moving speed of the subject is constant, the number of grid in the accessibility map can denote as time. In Figure 11, t0~t10 and T3~T10 are the subject moving grid number for different PIR sensing areas, the sum of them for the route1 is 211. For the A-start tracking algorithm, the iteration time can be set as 211. For the route2, the iteration time is 143.

**Figure 12:**
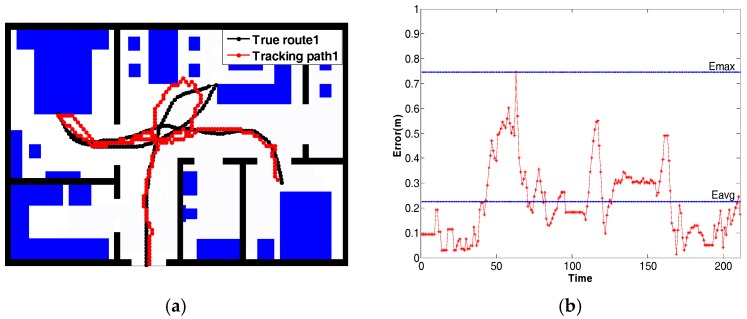
The result of position tracking with *True route1*. (**a**) The path estimated with *True route1*; (**b**) The error curve for *True route1*.

**Figure 13:**
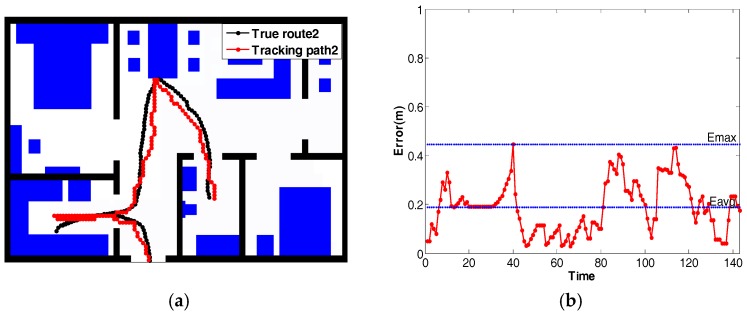
The result of position tracking with *True route2*. (**a**) The path estimated with *True route2*; (**b**) The error curve for *True route2*.

In Figure 12a, the localization and tracking results of a person following the pre-defined route1 are shown. The black line is the ground truth obtained from the OptiTrack system. The red line is the estimated trajectory. The distance error between the ground truth and the estimated lines is calculated for each node, as shown in Figure 12b. The maximum distance error is 0.747 m, and the minimum distance error is 0.021 m. On average, the mean of the distance error is 0.227 m. Another experiment was conducted using route2, shown in Figure 13. The mean of the distance error is 0.188 m. The maximum distance error is 0.445 m, and the minimum distance error is 0.109 m. Besides, Table 2 shows the comparison between our method and some recent human tracking projects, in terms of average error, sensor number per m^2^, and overall accuracy.

## 4. Conclusions

In this work, we studied the problem of human localization in an indoor environment. We have proposed a method that combines a grid-based accessible map and an A-star algorithm for human tracking. First, the grid-based accessibility map, which reflected human visiting preferences and the physical layout of the area is built. Then PIR sensors which are deployed according to the grid-based accessibility map provide an external rough position of the human. Finally, the A-star algorithm fuses the PIR sensor data and grid-based accessible map information to estimate the trajectory and reduce the errors. In our research, PIR sensor data are collected and sent through a Zigbee communication unit to a PC. The experiment results demonstrate the performance of our method. Our method can be used for robots who accompany the elderly who are living alone, help the robots understand the environment better and supply better service by localization tracking.

In the future, we will further improve our algorithm by considering more factors. For example, we need to consider the speed and direction of human movement in smart homes. More practical PIR sensors should also be considered, especially with regard to the response delay. We will also investigate tracking multiple residents living together in a smart home. 

## Figures and Tables

**Table 1:** PIR sensor parameters.

Parameter	Value
No. of PIR sensors	10
Number of PIR sensing area	14
Maximum coverage ratio $c_{\max}$	0.4182
Actual coverage rate $c_{act}$	0.3903
Overlap rate oc	0.0714

**Table 2:** Recent project of human tracking.

Method	Average Error (m)	Num_Uint (/m^2^)	Text Bed
[19]	0.85	0.017	96%
[20]	0.20	0.750	96.21%
[21]	0.18	0.673	86.21%
Our method	0.21	0.116	87.36%
